# HgCdTe mid-Infrared photo response enhanced by monolithically integrated meta-lenses

**DOI:** 10.1038/s41598-020-62433-w

**Published:** 2020-04-14

**Authors:** Fangzhe Li, Jie Deng, Jing Zhou, Zeshi Chu, Yu Yu, Xu Dai, Huijun Guo, Lu Chen, Shangkun Guo, Mengke Lan, Xiaoshuang Chen

**Affiliations:** 1 State Key Laboratory of Infrared Physics, Shanghai Institute of Technical Physics, Chinese Academy of Sciences Shanghai, 200083 China; 2 University of Chinese Academy of Sciences, Beijing, 100049 China; 3 School of Physical Science and Technology, ShanghaiTech University, Shanghai, 201210 China; 4 Key Laboratory of Infrared Imagining Material and Detectors, Shanghai Institute of Technical Physics, Chinese Academy of Sciences Shanghai, 200083 China

**Keywords:** Mid-infrared photonics, Optoelectronic devices and components, Metamaterials

## Abstract

Polarization-independent dielectric meta-lens is proposed to monolithically integrate with a HgCdTe infrared photodetector to concentrate power flux into a reduced photosensitive area for performance enhancement. Although a reduction in photosensitive area could suppress the dark current, the more seriously reduced light absorptance would degrade the specific detectivity D^*^. The integration of the meta-lens could reverse the situation by improving the absorptance of the photosensitive region. The meta-lens composed of an array of nano-pillars with varying diameters is formed by carving the CdZnTe substrate of the HgCdTe detector so that the integration could be accomplished *in situ*. The meta-lens focuses the incident light through the CdZnTe medium and at the HgCdTe photosensitive region. The focal spot is about the wavelength size and the focusing efficiency is above 63%. Concerning a HgCdTe detector with a pitch size of 40 μm × 40 μm, when the photosensitive area is reduced to 5 μm × 5 μm, the meta-lens could still keep the light absorptance above 50%, which is 49 times higher than that of the device without the meta-lens. The dark current reduces with the decreasing photosensitive area in a linear manner. When the photosensitive area shrinks from 40 μm × 40 μm to 10 μm × 10 μm or 5 μm × 5 μm, the dark current reduces by 16 or even 64 times. Compared to the pristine device, the employment of the meta-lens together with the reduction in photosensitive area could enhance D^*^ by 5.5 times for the photosensitive area as 5 μm × 5 μm. Further, the meta-lens exhibits a good dispersion tolerance over the wavelength range from 3.3 μm to 5 μm. The averaged detectivity enhancement over this spectrum range is around 3 times for the photosensitive area as 5 μm × 5 μm. The angular response of the meta-lens integrated detector depends on the focal length. For a focal length of 73 µm or 38 µm, the angle of view for a 5 μm × 5 μm photosensitive area is 4.0° or 7.7°. For the inter-pillar distance to be 2 µm in our design, the influence of the coupling effect between the nano-pillars on the performance of the meta-lens is little.

## Introduction

Infrared photodetectors are widely employed in a lot of science and technical fields including remote sensing, meteorological monitoring, military target detection and biomedical sensing^1^. In order to fulfil the quickly increasing demands, high-performance infrared detectors with high responsivity and low dark current are eagerly pursued. Concerning those infrared photodetection materials with large absorption coefficients and high quantum efficiencies, such as HgCdTe, the incident photons are almost completely turned into electronic signals so that further increasing the responsivity is difficult. Then, reducing the active volume to lower the dark current and at the same time remaining the light collection efficiency through light concentration methods becomes a promising way to enhance the detectivity. In this direction, the integration of micro-lens with infrared photodetectors has attracted much attention^2–9^. However, limited numerical aperture, complicated packaging and alignment, and the difficulty in curved surface formation impede the practical application in infrared photodetection^10–12^. Recently, the quickly developing meta-surface based flat optics provide us a new approach to concentrate light into a reduced active volume. Meta-surface has been successfully applied to control the phase, amplitude and polarization of the incident light by altering the shape and the size of each meta-unit and arranging them a proper way. In a wide range of spectrum from the visible to the terahertz band^13–24^, many optical applications based on meta-surfaces have been realized and reported, including meta-surface lenses (meta-lenses), waveplates, holograms and polarimeters^25–32^. The ultra-compact sizes of the flat optics make them promising to substitute conventional ones in some situations. Among all the flat optics, meta-lenses received the most research interest due to the broad application areas and the distinct advantages, such as self-alignment during fabrication, high numerical apertures^33,34^, and solid-immersion type of integration^35^. Meta-lenses have been demonstrated to be effective to focus the incident light through a high-index semiconductor^35^, indicating that the monolithic integration of a meta-lens into a semiconductor based infrared photodetector for light concentration in a reduced active volume is promising and feasible.

In this work, we propose to monolithically integrate a dielectric transmissive and polarization-independent meta-lens into a mesa-type HgCdTe infrared photodetector to enhance light collection in a reduced active volume for detectivity enhancement. The meta-lens is based on the all-CdZnTe meta-surface, which could be directly fabricated on the backside of the HgCdTe infrared detector by standard photolithography and etching process. For mid-infrared light with a wavelength around 4 μm, the meta-lens with a pitch size of 40 μm can focus the incident light into a small spot of the wavelength size on the photosensitive area with a full-width at half-maximum (FWHM) of 4 μm. With the help of the meta-lens, the absorptance of the photosensitive region remains above 50%, even though the photosensitive area is reduced by 64 times to 5 μm × 5 μm. The specific detectivity is enhanced by 5.5 times compared to the reference device with no reduction in photosensitive area and with no meta-lens. Further, the composite device exhibits a good dispersion tolerance over the wavelength range from 3.3 μm to 5 μm, and the averaged detectivity enhancement is around 3 times. The angular response of the meta-lens integrated infrared detector is analyzed. And the coupling effect between the nano-pillars is discussed. We envision that the monolithically integrated meta-lens is promising to improve the sensitivity of the traditional semiconductor infrared photodetectors.

## Device structure

**Figure 1:**
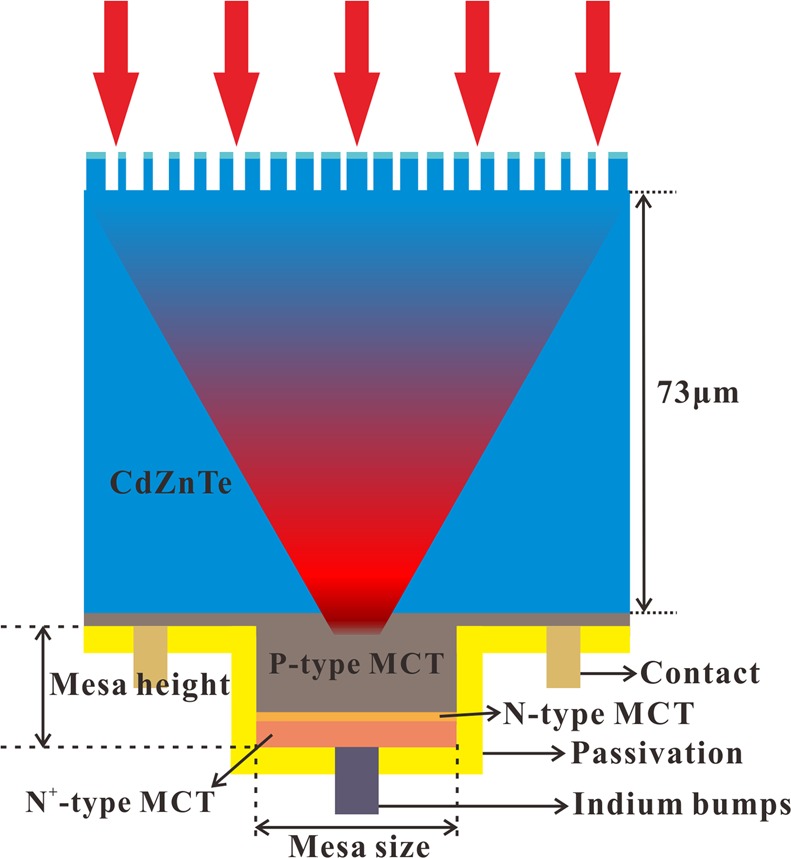
Schematic diagram of the meta-lens integrated HgCdTe infrared detector. The meta-lens forms at the top by etching into the CdZnTe substrate. The infrared incident light is focused through the CdZnTe substrate and onto the HgCdTe active region, which consists of P, N, N^+^ type of HgCdTe. The CdZnTe substrate has a height of 73 μm. The contact, passivation and indium bumps are also presented.

The integration of such a meta-lens with a mesa-type HgCdTe detector is schematically illustrated in Fig. 1. The HgCdTe detector is actually a single pixel of a focal plane array and ready for flip-chip bonding. The meta-lens consisting of a nano-pillar array is proposed to be fabricated by hard-mask-assisted reactive ion etching^36–41^. The hard mask made of SiO_2_ can enhance the etching selectivity by over 37 times^38^ compared to a photoresist mask, and then enable the formation of high-aspect-ratio structures^39–41^. In this case, each nano-pillar contains a SiO_2_ cap on the top. By choosing a proper thickness of this mask layer, the transmission through the meta-lens is enhanced. The focal length *f* is determined by the thickness of the CdZnTe film, which typically varies from 30 μm to 800 μm^42^, and it is assumed to be 73 μm in this study without loss of generality. The HgCdTe layer is epitaxially grown on the other side of the CdZnTe. The active area is defined by the mesa. As the mesa size decreases, a lower dark current is expected. In order to remain the light collection efficiency, the incident light is desired to be focused into the reduced active region. As a photovoltaic HgCdTe infrared photodetector, the mesa contains P, N and N^+^ regions. The thicknesses and doping levels of each region are set to be 9.96 μm, 0.43 μm, 2.5 μm and 8.4 × 10^15^ cm^−3^, 2 × 10^16^ cm^−3^, 2.4 × 10^17^ cm^−3^, respectively, as typical values for HgCdTe photodetectors operating in the mid-infrared range^43^. The mesa height is around 10 μm. For the numerical simulations, the wavelength dependent refractive index of CdZnTe follows ref. ^44^. and that of SiO_2_ follows refs. ^45,46^. The composition of the Hg_*1-x*_Cd_*x*_Te is set to be *x* = 0.276 for mid-infrared detection^47^, and the corresponding complex refractive index are obtained through refs. ^48,49^.

**Figure 2:**
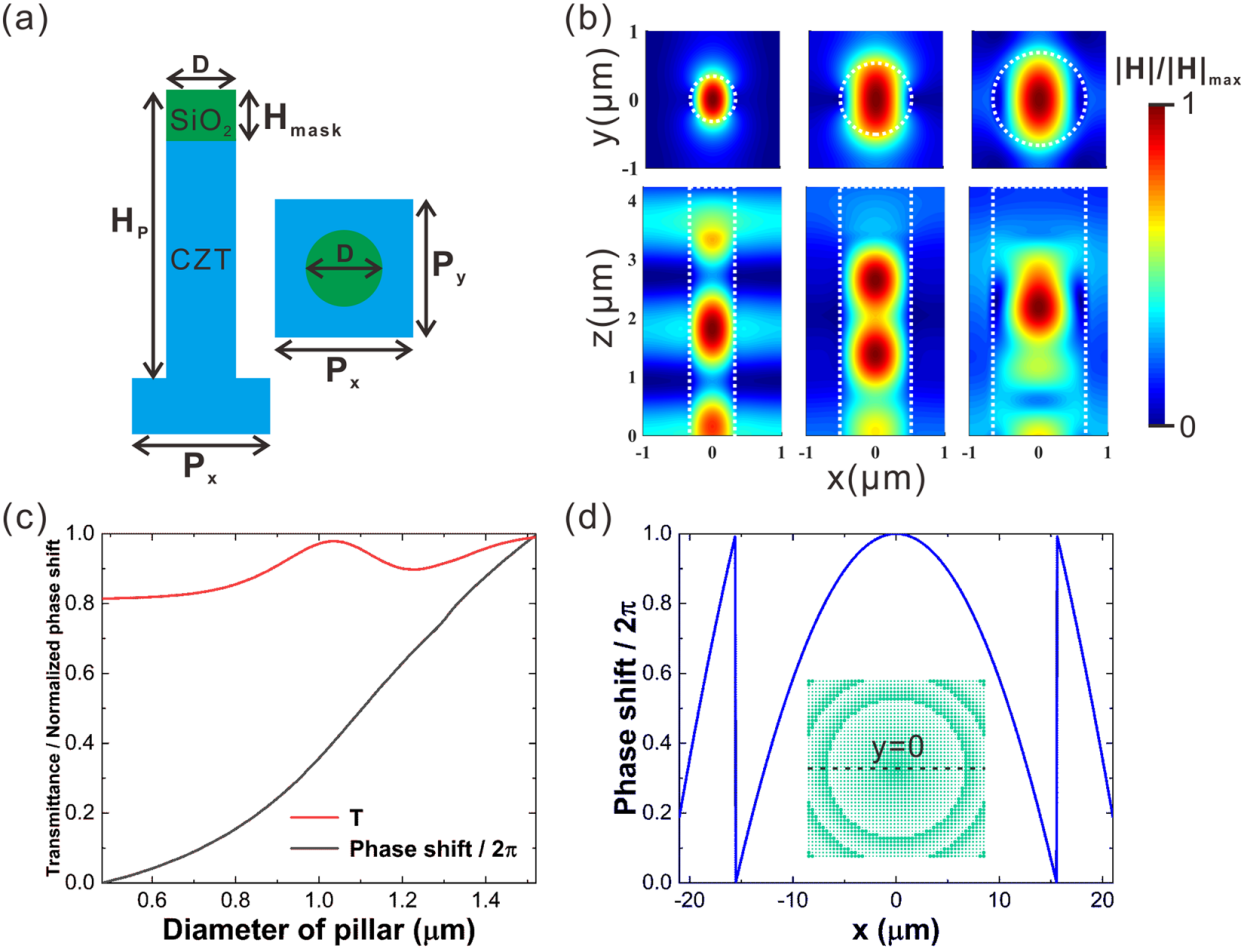
(**a**) Sketch of the meta-lens unit cell. *P*_x_ = *P*_y_ = 2 μm, *H*_p_ = 4.23 μm, *H*_mask_ = 0.73 μm, *D* varies from 0.69 μm to 1.52 μm. **(b)** Distributions of the normalized magnetic field on the (*x*, *y*) and (*x*, *z*) cross sections of the nano-pillars with three different diameters (*d* = 0.69 μm, 1.1 μm and 1.52 μm). A plane wave with a wavelength of 4 μm and polarized in the *x*-direction is normally incident on the nano-pillars. The boundaries of the pillar are depicted by dashed white lines. **(c)** Transmittance and normalized phase shift of the unit cell as a function of the diameter of the nano-pillar. **(d)** Required analog phase distribution along the *x*-axis cutting through the center of the meta-lens. Inset: schematic of the meta-lens. Two different colors (green and white) are used to distinguish the pillars from the substrate.

Concerning a plane wave normally incident on the detector, the meta-lens on the top would focus the light at the interface between the CdZnTe and the HgCdTe. As illustrated in Fig. 2[a], the meta-lens is composed of an array of CdZnTe nano-pillars. The nano-pillars are formed by carving the CdZnTe substrate, which also supports the epitaxially grown HgCdTe on the other side. The circular cross section is set for polarization-independent response. Each unit cell of the meta-lens occupies a 2 μm × 2 μm square area and contains one nano-pillar at the center. All the nano-pillars are of the same height (*H*_p_ = 4.23 μm and *H*_mask_ = 0.73 μm), but vary in diameter according to the positions. Each nano-pillar works like a waveguide for the incident light. The dispersion relation of the waveguide mode is dependent on the diameter. As shown in Fig. 2[b], the phase of the waveguide mode advances faster in a thicker nano-pillar. Therefore, by finely controlling the diameters and the inter-distance of the nano-pillars, the outgoing phase at each unit cell could be tuned over the range from 0 to 2π while the transmittance could remain similar (Fig. 2[c]), so that the wave front of the light passing through the meta-surface could be freely designed. Ideally, the phase distribution at the plane just after a convergent lens should follow an inward propagating spherical wave, as described by

1 $$\varphi (x,y)=-\frac{2\pi }{{\lambda }_{d}}\cdot {n}_{s}\cdot (\sqrt{{x}^{2}+{y}^{2}+{f}^{2}}-f),$$

where *λ*_*d*_ denotes the operating wavelength, *f* the focal length and *n*_*s*_ the refractive index of the medium. When the phase distribution of the light just passing through the nano-pillar array follows Eq. 1, the light field would converge into a spot, and the nano-pillar array just functions like a lens. For the incident wavelength of 4 μm, with the diameter *d* of the nano-pillar varying from 0.69 μm to 1.52 μm, a phase shift over 2π could be achieved, and the transmittance averagely reaches 90% (Fig. 2[c]). Figure 2[d] presents the required phase distribution *φ*(*x*, 0) along the *x*-axis cutting through the center of the meta-lens for the incident light to be focused at a plane 73 μm away from the meta-surface inside the CdZnTe substrate. As illustrated in the insert of Fig. 2[d], the meta-lens contains 21 × 21 unit cells. The nano-pillar diameters are optimized according the evaluation function $C=|{T}_{m}{e}^{i\varphi (x,y)}-T(d){e}^{i\varphi (D)}|$, which concerns both phase and transmittance^50^. For a nano-pillar at the position (*x*, *y*), an appropriate diameter *D*, which minimizes *C*, is selected. *φ(D)* and *T(D)* are the phase shift and the transmittance at the meta-element (*x*,*y*) with a nano-pillar of diameter *D*. *T*_*m*_ is the averaged transmittance over all the meta-elements. The finite difference time domain method is employed to simulate the optical behavior of this device, and the simulation regime consists of three regions: the air, the CdZnTe substrate, and the HgCdTe active region. The electrical properties of the infrared detector are investigated through the technology for computer-aided design (TCAD).

## Result and analysis

**Figure 3:**
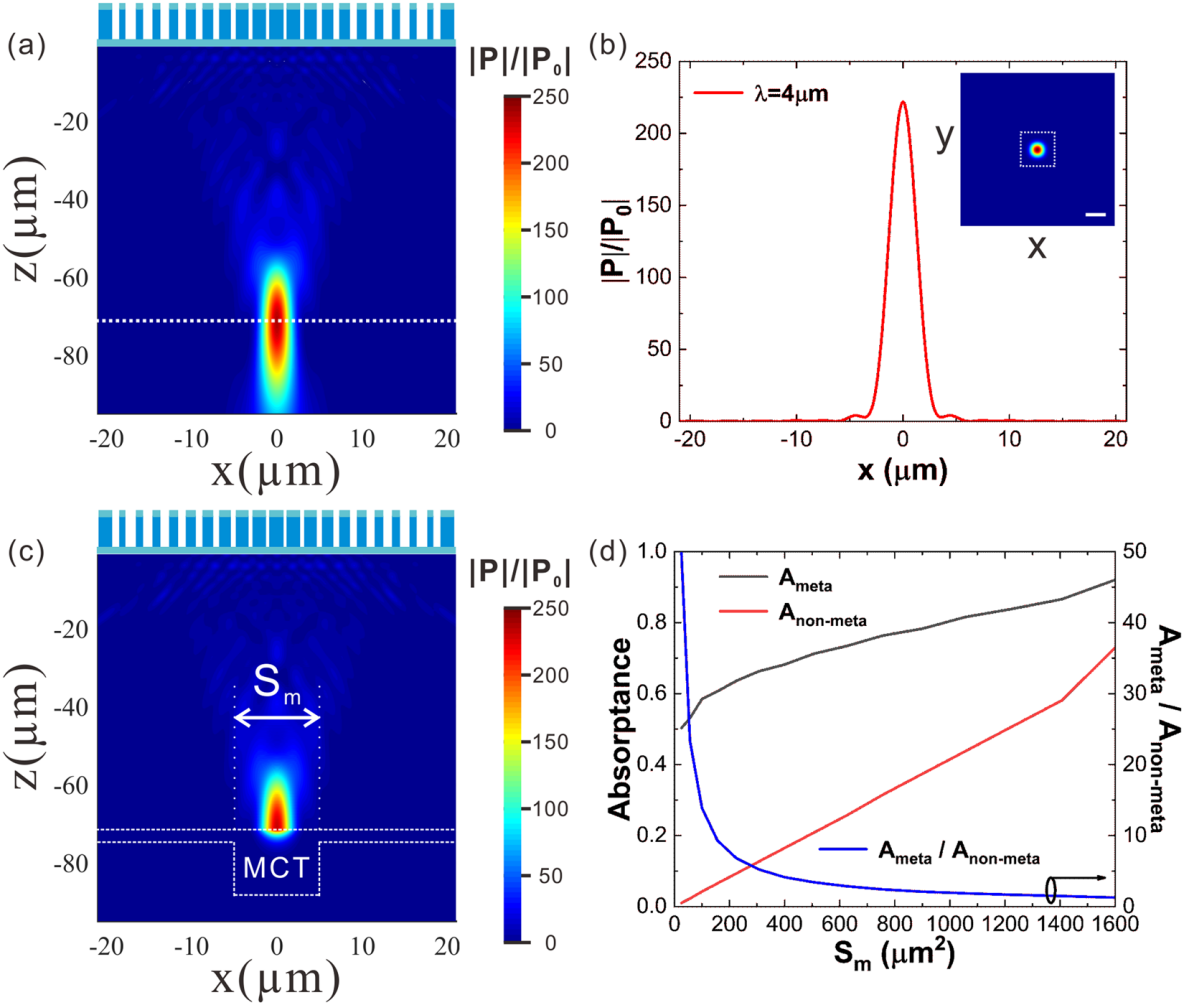
(**a**) Relative power flux |*P*|/|*P*_0_|on the *x*-*z* cross-section of the CdZnTe substrate (without HgCdTe active region) cutting through the center of the meta-lens. **(b)** |*P*|/|*P*_0_|profile along the dashed line marked out in **(a)**. Inset: |*P*|/|*P*_0_|distribution on the *x*-*y* cross section cutting through the focal spot. Scale bar: 5 μm. **(c)** |*P*|/|*P*_0_|distribution on the *x*-*z* cross section of the full structure of the meta-lens integrated HgCdTe infared detector. The active region is sketched out the outline by dashed white lines. **(d)** Absorptance of the active region with (*A*_*meta*_) or without (*A*_*non-meta*_) meta-lens at various photosensitive areas. And the absorption enhancement by the meta-lens (*A*_*meta*_*/A*_*non-meta*_).

Figure 3[a,b] present how the incident light is focused into the CdZnTe substrate by the meta-lens. Figure 3[a] presents the distribution of the relative power flux (|*P*|/|*P*_0_|) on the *x-z* cross section cutting through the center of the CdZnTe supported meta-lens. It is exhibited that the incident light is focused at a plane about 73 μm below the meta-lens inside the CdZnTe. The focal spot has a full width half maximum (FWHM) of 4 μm, as revealed by the relative power flux (|*P*|/|*P*_0_|) profile (Fig. 3[b]) along the dashed line in Fig. 3[a]. The concentrated light power at the center of the focal spot is 235 times higher than the incident power. The focusing efficiency, defined as the ratio of light power entering a square of 4 × FWHM centered at the focal spot on the *x*-*y* plane (dashed frame in the inset of Fig. 3[b]) to the total power incident on the meta-lens^50^, is above 63%. Figure 3[c] illustrates a full structure simulation of the meta-lens integrated HgCdTe detector. The incident light is successfully converged at the photosensitive region and it is quickly absorbed after penetrating into the HgCdTe. As shown in Fig. 3[d], with the help of the meta-lens, the absorptance of the photosensitive region (*A*_*meta*_) remains higher than 50% for the photosensitive area larger than 5 μm × 5 μm, while in the absence of the meta-lens the absorptance of the photosensitive region (*A*_*non-meta*_) degrades with the decreasing area almost linearly, indicating that the meta-lens could drastically enhance the absorptance for reduced photosensitive areas. Specifically, the meta-lens improves the absorptance by 49 times or 13 time for *S*_m_ = 5 μm × 5 μm or *S*_m_ = 10 μm × 10 μm.

**Figure 4:**
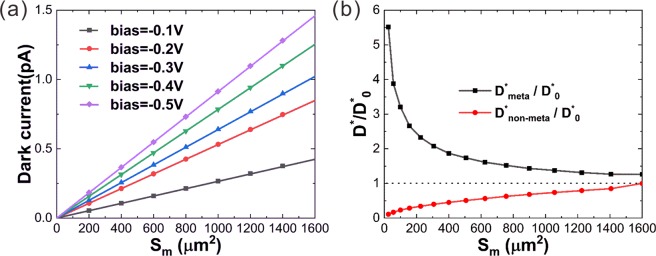
(**a**) Dark currents as a function of the photosensitive area at different bias voltages from −0.5 V to −0.1 V. **(b)** The relative detectivity (D*/D*_0_) as a function of the photosensitive area for the detector with or without the meta-lens.

An infrared photodetector is evaluated not only by the photoresponse but also by the noise. For such a HgCdTe photovoltaic detector, the photoresponse is proportional to the light absorptance of the active region, and the noise is largely decided by the dark current^51^. Figure 4[a] presents the dark current (*I*_d_) as a function of the photosensitive area (*S*_m_) at typical operating bias voltages from −0.1 V to −0.5 V. The dark current reduces with the decreasing photosensitive area in a linear manner. When the photosensitive area shrinks from 40 μm × 40 μm to 10 μm × 10 μm or 5 μm × 5 μm, the dark current reduces by 16 or even 64 times. The reduction in dark current would steadily suppress the noise (*I*_n_) as favored by infrared detectors. However, in the absence of the meta-lens, the light absorptance of the photosensitive region and hence the responsivity *R*_i_ also decrease with *S*_m_ linearly. In this case, the detectivity ${D}^{\ast }={R}_{i}\sqrt{S\cdot \Delta f}/{I}_{n}$ as a figure of merit for an infrared detector degrades with the decreasing *S*_m_ (Fig. 4[b] red line), because the responsivity *R*_i_ is proportional to *S*_m_ while the noise current ${I}_{n}=\sqrt{2e{I}_{d}\Delta f}$ is proportional to the square root of *S*_m_^52^. *S* stands for the pitch area of the HgCdTe detector, which remains 40 μm × 40 μm in our case. Δ*f* denotes the bandwidth. *e* is the electron charge. With the help of the meta-lens, the absorptance of the photosensitive region and hence the responsivity is significantly improved (Fig. 3[d]). As a result, D^*^ is prominently enhanced with the decreasing *S*_m_, as shown in Fig. 4[b]. D^*^_0_ denotes the detectivity of the HgCdTe detector with no reduction in the photosensitive area and with no meta-lens. The detectivity enhancement as represented by D^*^_meta_/D^*^_0_ is 3.2 for *S*_m_ = 10 μm × 10 μm and it reaches 5.5 for *S*_m_ = 5 μm × 5 μm, indicating that the integration of the meta-lens in an infrared detector to focus light onto a reduced photosensitive area could promisingly enhance the device performance.

**Figure 5:**
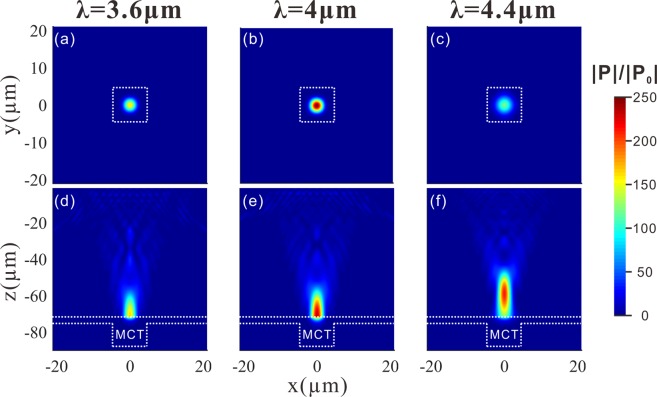
(**a–c**) Relative power flux (|*P*|/|*P*_0_|) distributions on the x-y cross-section cutting through the focal spot for different incident wavelengths (λ = 3.6 μm, 4 μm, 4.4 μm). **(d-f)** Relative power flux (|*P*|/|*P*_0_|) distributions on the *x*-*y* cross-section cutting through the focal spot for different incident wavelengths corresponding to **(a–c)**. The active region is sketched by the dashed frame.

Although the meta-lens is not intentionally designed to be achromatic, it could still effectively enhance the detectivity of the HgCdTe detector over the wavelength range from 3.3 μm to 5 μm, as benefited from the elongation of the focal spot in the *z*-direction. The meta-lens is designed for the wavelength of 4 μm. At this specific wavelength, the incident light could be perfectly focused onto the photosensitive region and directly at the interface between the CdZnTe and the HgCdTe, as shown in Fig. 5[a-f]. When the wavelength deviates from 4 μm, the focal plane moves downwards or upwards due to the dispersion of the meta-lens^53^. Consequently, the field concentration at the interface between the photosensitive region and the CdZnTe substrate becomes less tight so that a part of power leaks out, leading to reduction in absorptance and responsivity.

**Figure 6:**
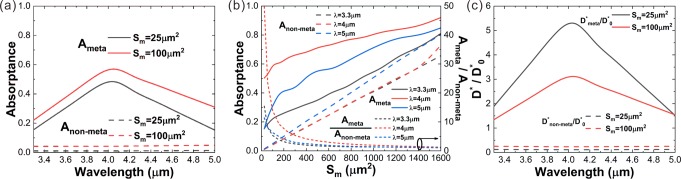
(**a**) Absorptance by the active region of the HgCdTe detector with or without the meta-lens at different incident wavelengths. Two different photosensitive areas (S_m_ = 5 μm × 5 μm and S_m_ = 10 μm × 10 μm) are investigated. **(b)** Absorptance by the active region of the HgCdTe detector with (*A*_*meta*_) or without (*A*_*non-meta*_) meta-lens at various photosensitive areas for three different wavelengths. And the absorption enhancement by the meta-lens (*A*_*meta*_*/A*_*non-meta*_). **(c)** The relative detectivity (D*/D*_0_) versus incident wavelength for two photosensitive areas (25 μm^2^ and 100 μm^2^).

As shown in Fig. 6[a], the absorptance of the photosensitive region reaches a maximum at the designed wavelength (4 μm) and reduces when the wavelength either decreases or increases. Fortunately, since the focal spot is elongated in the *z*-direction as shown in Fig. 5, the offset between the focal plane and the surface of the photosensitive region does not severely degrades the absorptance. For *S*_m_ = 5 μm × 5 μm, *A*_meta_ remains higher than 15.2% over the range from 3.3 μm to 5 μm and the averaged *A*_meta_ reaches 27%. For *S*_m_ = 10 μm × 10 μm, *A*_meta_ remains higher than 22% over the same wavelength range and the averaged *A*_meta_ reaches 37%. Therefore, the averaged enhancement of the absorptance over the wavelength range from 3.3 μm to 5 μm is around 24.5 times for *S*_m_ = 5 μm × 5 μm and it is around 8.5 times for *S*_m_ = 10 μm × 10 μm (Fig. 6[b]). Concerning the detectivity, the averaged enhancement (D^*^_meta_/D^*^_0_) is around 3 times for *S*_m_ = 5 μm × 5 μm and 2 times for *S*_m_ = 10 μm × 10 μm (Fig. 6[c]). The good chromatic dispersion tolerance indicates that the integration of the meta-lenses and the infrared photodetectors for performance enhancement is prospective.

**Figure 7:**
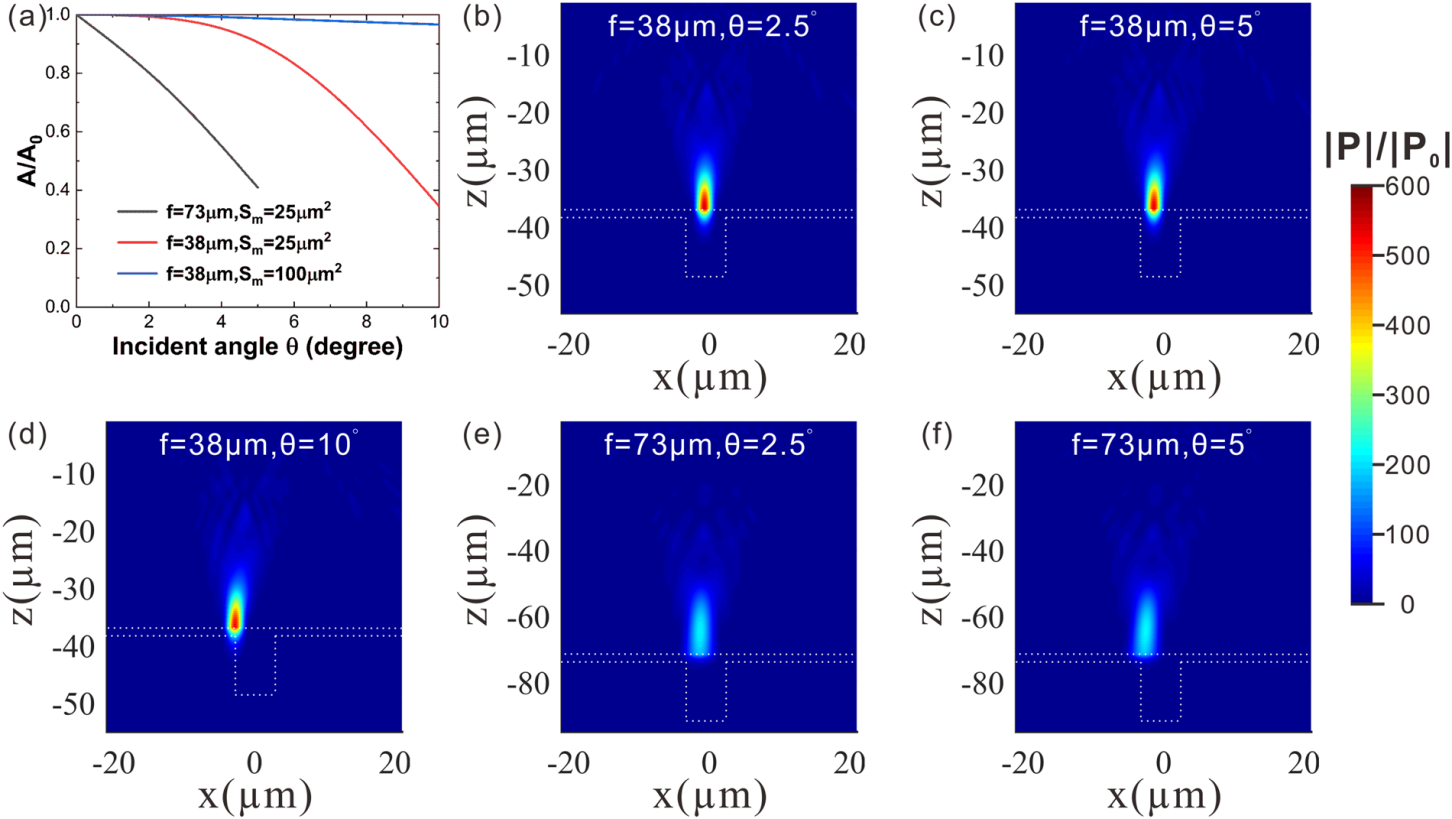
(**a**) Normalized active region absorptance as a function of incident angle. Black line: focal length 73 μm, active region area 5 μm × 5 μm. Red line: focal length 38 μm, active region area 5 μm × 5 μm. Blue line: focal length 38 μm, active region area 10 μm × 10 μm. **(b-d)** Power flux distributions on the *x*-*z* cross section (*y* = 0 μm) of the meta-lens integrated HgCdTe detector for the incident angles of 2.5°, 5°, 10°. The focal length *f* of the meta-lens is 38 μm. θ denotes the incident angle. **(e-f)** Power flux distributions on the *x*-*z* cross section (*y* = 0 μm) of the meta-lens integrated HgCdTe detector for the incident angles of 2.5°, 5°. The focal length of the meta-lens is 73 μm.

Moreover, the angular response of the meta-lens integrated infrared detector is important since sensing angular light is somehow demanding in real applications. Concerning the meta-lens with a focal length of 73 μm, the absorptance of the active region (5 μm × 5 μm) drops by more than a half when the incident angle increases to 5° (Fig. 7[a] black line). This degradation also occurs for traditional microlens^2,54^. Shortening the focal length is a method to alleviate this degradation. As shown in Fig. 7[a] red line, when the meta-lens is designed to have a short focal length of 38 μm, the absorptance of the active region drops much more slowly than that for the long focal length of 73 μm. At the 5° oblique incidence, the active region absorptance for the short-focal-length case remains 94% of that at normal incidence. This effect is also illustrated by the distribution of the power flux (Fig. 7[b–f]). The focal spot in the short-focal-length case offsets more slowly with the incident angle than that in the long-focal-length case. Concerning focal length adjustment, especially realization of short focal lengths, meta-lenses have an advantage over traditional micro-lenses. Shortening the focal length of a meta-lens is simply to rearrange the distribution of the nano-pillars, while shortening the focal length of a traditional micro-lenses needs to enlarge the surface curvature, which takes more efforts in fabrication. Another method to alleviate the degradation with incident angle is to enlarge the active region. As shown in Fig. 7[a], when the active region is enlarged to 10 μm × 10 μm, the absorptance of the active region remains higher than 98% of that at normal incidence over for oblique incidence up to 10°. The angle of view (AOV) is defined as 2arctan(*s*/(2 *f*))^2^, where *s* is the size of the active area, and *f* is the focal length. For *s* = 5 μm and *f* = 73 μm, the AOV = 4.0°. For *s* = 5 μm and *f* = 38 μm, the AOV = 7.7°. For *s* = 10 μm and *f* = 38 μm, the AOV = 15.4°.

**Figure 8:**
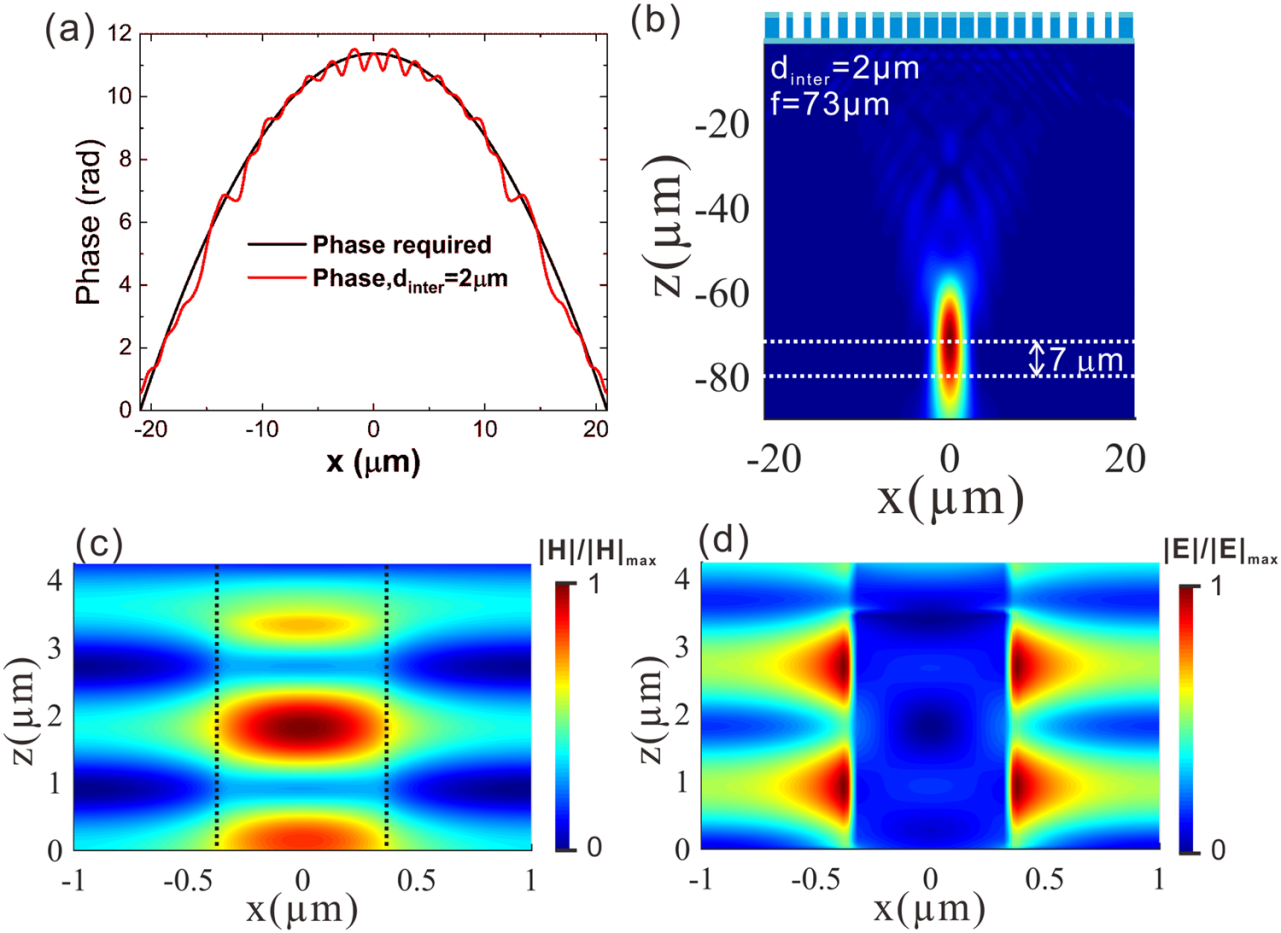
(**a**) Designed phase distribution profile of the meta-lens based on unit cell simulation (black line) and the phase distribution profile based on full meta-lens simulation with the center-to-center inter-pillar distance as 2 μm (red line). **(b,c)** Power flux distributions on the *x*-*z* cross section (*y* = 0 μm) of the meta-lens integrated CdTeZn. The meta-lense is designed to have a focal length of 80 μm. **(c-d)** *H* and *E* field distributions on the *x*-*z* cross section of a unit cell with the diameter of the nano-pillar as 0.69 μm. The center-to-center inter-pillar distance is 2 μm.

Another important issue for the meta-lens is the coupling between the nano-pillars. A metalens is a nonperiodic structure. However, the design of the nano-pillars is based on unit cell simulation employing periodic boundary conditions. The coupling between the nano-pillars affects the propagation constant of the waveguide mode and hence the phase accumulation. Since a nano-pillar in the meta-lens feels a different environment than in the unit cell simulation, the phase distribution over the meta-lens deviates from the design. In this work, the unit cell simulation with periodic boundary conditions is only used as the reference for preliminary design. The full-meta-lens as a nonperiodic structure is finally simulated to evaluate the performance of real device. It is true that there is deviation between the unit cell simulation and the full-meta-lens simulation. But, the deviation is small. As confirmed by Fig. 8[a], the phase distribution profile based on full meta-lens simulation (red line) deviates from the design based on unit cell simulation (black line). For the center-to-center inter-pillar distance (*d*_inter_) as 2 μm, the focal length is about 73 μm, shorter than the designed value of 80 μm (Fig. 8[b]). The deviation is only 8.8%. The coupling between the nano-pillars is also revealed by the field distribution. As shown in Fig. 8[c,d], the light field in the space between the nano-pillars is obvious.

Although traditional micro-lens arrays have been applied to enhance the light coupling efficiency for photodetector since a long time ago^55^, meta-lenses have several potential advantages over traditional micro-lenses. Meta-lenses could achieve higher aspect ratio^33,34^ than traditional micro-lenses^10,11^, and a higher aspect ratio corresponds to a big angle tolerance. Meta-lenses do not need complicated packaging and alignment since they are self-aligned once fabricated. Moreover, they are more compatible to flip-chip-bonded infrared focal plane arrays since they can be fabricated directly on the substrate for a solid-immersion type of focusing^35^. And meta-lenses can be achromatic by properly designing the multiple resonances of the unit-cells^56^, so that they are more suitable for broadband applications potentially. A more detailed comparison can be found in the Supplementary Material.

## Conclusion

In conclusion, a transmissive polarization-independent dielectric meta-lens is proposed to monolithically integrate with a HgCdTe infrared photodetector to concentrate power flux into a reduced photosensitive area for performance enhancement. The whole device unit can be regarded as a pixel element of focal plane, so it can be used for the integrated preparation of FPA. The meta-lens composed of an array of nano-pillars with varying sizes is formed by carving the CdZnTe substrate of the HgCdTe detector and an SiO_2_ layer is added for higher transmission. A meta-lens integrated HgCdTe infrared photodetector with a pitch size of 40 μm × 40 μm was numerically investigated. Although a reduction in photosensitive area could suppress the dark current, the more seriously reduced light absorptance would degrade the specific detectivity D*. With the help of the meta-lens, which focuses the light into a spot of wavelength size with an efficiency over 63%, the light absorptance of a 5 μm × 5 μm or 10 μm × 10 μm photosensitive region still remains 50% or 58% although the photosensitive area is reduced by 64 or 16 times compared to the pristine. Consequently, the integration of the meta-lens enhances the D* by 49 or 14 times for *S*_m_ = 5 μm × 5 μm or *S*_m_ = 10 μm × 10 μm. Compared to the pristine device, the integration of the meta-lens together with the reduction in photosensitive area enhances the D^*^ by 5.5 or 3.2 times for *S*_m_ = 5 μm × 5 μm or *S*_m_ = 10 μm × 10 μm. Further, the meta-lens exhibits a good dispersion tolerance over the wavelength range from 3.3 μm to 5 μm. The averaged detectivity enhancement over this spectrum range is around 3 times for *S*_m_ = 5 μm × 5 μm and 2 times for *S*_m_ = 10 μm × 10 μm. The angular response of the meta-lens integrated detector depends on the focal length. For a focal length of 73 μm, the AOV for a 5 μm × 5 μm photosensitive area is 4.0°. When the focal length is reduced to 38 μm, the AOV for a 5 μm × 5 μm photosensitive area increases to 7.7° and it reaches 15.4° for a 10 μm × 10 μm photosensitive area. For the inter-pillar distance to be 2 μm in our design, the influence of the coupling effect between the nano-pillars on the performance of the meta-lens is little. Therefore, the monolithic integration of a meta-lens provides us a promising way to enhance the performance of infrared photodetectors and even focal plane arrays.

## Methods

The performance of the proposed metalens are characterized by using the three-dimensional finite difference time domain (FDTD) method from Lumerical Inc^57^. For the simulation of the unit cell, periodic boundary conditions are applied along the x and y axis and perfectly matched layers (PML) is applied along the z axis. For the simulation of the metalens, PML are applied along the three axis for the specific phase elements of the designed metalens. The simulated total area of the metalens is 40 × 40 μm^2^ with 21 × 21 unit cells. Substrate height of CdZnTe is chosen to be 73 μm. The electrical properties of the device unit shown are simulated by Sentaurus TCAD. Thicknesses and doping levels of each region (P,N,N^+^) are set to be 9.96 μm, 0.43 μm, 2.5 μm and 8.4 × 10^15^ cm^−3^, 2 × 10^16^ cm^−3^, 2.4 × 10^17^ cm^−3^, respectively.

## Supplementary information

Supplementary information.
