# Supplementary material for: HgCdTe mid-Infrared photo response enhanced by monolithically integrated meta-lenses
Source: Sci Rep. 2020 Apr 14;10:6372. doi: 10.1038/s41598-020-62433-w (PMC7156491; doi:10.1038/s41598-020-62433-w)
Supplement: Supplementary file 1 — Supplementary information. [file 41598_2020_62433_MOESM1_ESM.docx]

**HgCdTe mid-Infrared photo response enhanced by monolithically integrated meta-lenses**

**(Supplementary Information)**

Fangzhe Li^1,2,3^, Jie Deng^1,2^, Jing Zhou^1^, Zeshi Chu^1,2^, Yu Yu^3^, Xu Dai^1,2^, Huijun Guo^4^, Lu Chen^4^, Shangkun Guo^1,2^, Mengke Lan^1^, Xiaoshuang Chen^1,3^

^1^State Key Laboratory of Infrared Physics, Shanghai Institute of Technical Physics, Chinese Academy of Sciences, Shanghai 200083, China;

^2^University of Chinese Academy of Sciences, Beijing 100049, China;

^3^School of Physical Science and Technology, ShanghaiTech University, Shanghai 201210, China;

^4^Key Laboratory of Infrared Imagining Material and Detectors, Shanghai Institute of Technical Physics, Chinese Academy of Sciences, Shanghai 200083, China;

Correspondence and requests for materials should be addressed to J.Z. (email: [jzhou@mail.sitp.ac.cn](mailto:jzhou@mail.sitp.ac.cn) ) or X.C. (email: [xschen@mail.sitp.ac.cn](mailto:xschen@mail.sitp.ac.cn))

**Comparison between meta-lenses and microlenses for the purpose to enhance light coupling efficiency of photodetectors**

Meta-lenses have several potential advantages over traditional micro-lenses, concerning the application to enhance the light coupling efficiency for photodetectors, as listed below.

1. It is easier for meta-lenses than for micro-lenses to have high numerical apertures (N.A.) concerning the fabrication. A high numerical aperture corresponds to a short focal length and thus a large angle of view, which is favored for photodetector applications. In order to achieve a high numerical aperture, a big phase gradient is required. For meta-lenses, a big phase gradient can be realized by properly arranging the distribution of the unit cells. After the design is accomplished, the fabrication of a high N.A. meta-lens is as straightforward as that of a low N.A. meta-lens. For traditional micro-lenses, a big phase gradient requires a big curvature, which is difficult for fabrication. In practice, the numerical apertures of traditional micro-lenses are typically below 0.5 [1,2], while those of meta-lenses can approach 1 in air [3,4].
2. Although large-scale micro-lens array could be easily fabricated with grayscale lithography or self-assembly technique, the alignment between a micro-lens array and a photodetector array is not a simple task. This packaging step requires sophisticated instruments and great efforts, leading to high cost and low yield [5]. For meta-lenses, the structures are directly fabricated on the photodetector, so alignment is achieved naturally when the structures are made.
3. For infrared focal plane arrays, the flip-chip bonding with readout integrated circuits requires the incident light to go through the substrates of photosensitive materials. In this case, solid-immersion type of focusing is desired [6], and integrating meta-lenses with a focal plane array *in situ* by etching the substrate is a promising method. In comparison, integrating solid-immersion type of traditional micro-lenses with a focal plane array requires carving the substrate into a curved surface with a specific curvature, and this process is not as easy as making a meta-lens with all planar structures.
4. Traditional micro-lenses, as refractive optics, have chromatic aberration, while meta-lenses can be achromatic by properly designing the multiple resonances of the unit-cells [7]. Therefore, meta-lenses would be more suitable for broadband applications potentially.

In addition, the fabrication cost of the meta-lenses can be reduced by using large-scale nano-fabrication techniques, such as nano-imprint lithography [8].

**References:**

1. D. Wu, S. Wu, L. Niu, Q. Chen, R. Wang, J. Song, H. Fang, H. Sun, “High numerical aperture microlens arrays of close packing”, Appl. Phys. Lett. **97**, 031109 (2010).
2. A. Tripathi, T. Chokshi, N. Chronis, “A high numerical aperture, polymer-based, planar microlens array”, Opt. Express **17**, 19908 (2009).
3. R. Paniagua-Domínguez,. Y. Yu, E. Khaidarov, S. Choi, V. Leong, R. Bakker, X. Liang, Y. Fu, V. Valuckas, L. Krivitsky, A. Kuznetsov, “A Metalens with a Near-Unity Numerical Aperture”, Nano Lett. **18**, 2124 (2018).
4. H. Liang, Q. Lin, X. Xie, Q. Sun, Y. Wang, L. Zhou, L. Liu, X. Yu, J. Zhou, T. Krauss, J. Li, “An Ultra-high Numerical Aperture Metalens at Visible Wavelengths”, Nano Lett. **18**, 4460 (2018).
5. Ph Nussbaum, R. Volkel, H. Herzig, M. Eisner, S. Haselbeck, “Design, fabrication and testing of microlens arrays for sensors and microsystems”, Pure Appl. Opt. **6**, 617 (1997).
6. S. Zhang, A. Soibel, S. Keo, D. Wilson, Sir. B. Rafol, D. Ting, A. She, S. Gunapala, F. Capasso, “Solid-immersion metalenses for infrared focal plane arrays”, Appl. Phys. Lett. **113**, 111104 (2018).
7. S. Wang, P. Wu, V. Su, Y. Lai, M. Chen, H. Kuo, B. Chen, Y. Chen, T. Huang, J. Wang, R. Lin, C. Kuan, Tao Li, Z. Wang, S. Zhu, D. Tsai, “A broadband achromatic metalens in the visible”, Nature Nanotech. **13**, 227 (2018).
8. L. Jay Guo, “Nanoimprint Lithography: Methods and material requirements”, Adv. Mater. **19**, 495 (2007).
